# Efficacy and safety of different doses of vamorolone in boys with Duchenne muscular dystrophy: a systematic review and network meta-analysis

**DOI:** 10.3389/fneur.2024.1456559

**Published:** 2024-08-21

**Authors:** Qin Wang, Yaqing Zeng, Linna Jiao, Jianli He, Baoyi Li, Yihua Guo, Zhibin Song

**Affiliations:** Department of Neurology, Xiaolan People’s Hospital of Zhongshan, Zhongshan, China

**Keywords:** Duchenne muscular dystroph, vamorolone, glucocorticosteroids, prednisone, deflazacort

## Abstract

**Background and objectives:**

Several recent clinical studies have indicated that vamorolone is comparable in effectiveness to glucocorticosteroids for treating Duchenne muscular dystrophy (DMD). However, there is a lack of extensive data regarding the efficacy and safety of various doses of vamorolone. We conducted a study to evaluate the efficacy of different doses of vamorolone in boys with DMD, and compare the safety of vamorolone vs. glucocorticosteroids, prednisone or deflazacort in boys with DMD.

**Methods:**

We performed systematic searches of the PubMed, Embase, and Cochrane Library databases for vamorolone, glucocorticosteroids, prednisone or deflazacort in boys with DMD. We assessed statistical heterogeneity across trials based on the Newcastle Ottawa scale (NOS) tool test and I^2^ values, and mean differences were pooled using the random-effects model. We used traditional meta-analysis to evaluate efficacy and safety of vamorolone 6.0 mg/kg/d vs. vamorolone 2.0 mg/kg/d and vamorolone vs. prednisone. A network meta-analysis was applied to estimated the safety of vamorolone in comparison to glucocorticosteroids, prednisone and deflazacort. Our meta-analysis were performed using Revman 5.4 software, and our network meta-analysis were performed using Stata/MP 18.0.

**Results:**

In the meta-analysis, a total of 193 patients were analyzed across four clinical trials (97 patients receiving vamorolone 2 mg/kg per day; 96 patients receiving vamorolone 2 mg/kg per day). We observed that there were statistically significant differences in boys with DMD between vamorolone 6.0 mg/kg/d and vamorolone 2.0 mg/kg/d in TTSTANDV (MD = 0.03, 95%CI = 0.00–0.06, *p* = 0.04), TTRWV (MD = 0.13, 95%CI = 0.08–0.19, *p* < 0.01), 6MWT (MD = 24.54, 95%CI = 4.46–44.82, *p* = 0.02), TTCLIMBV (MD = 0.04, 95%CI = 0.01–0.06, *p* = 0.009), no significant difference in BMI z score (MD = 0.09, 95%CI = −0.03–0.20, *p* = 0.13). Indirect comparisons derived from network meta-analysis did not show significant differences among vamorolone, glucocorticosteroids, prednisone and deflazacort in BMI z score.

**Conclusion:**

Our findings implied that boys with DMD who took vamorolone 6 mg/kg daily instead of 2 mg/kg daily may be safer and have superior motor function. However, more large sample randomized controlled trials are needed to confirm our results.

**Systematic Review Registration:**

This systematic review and meta-analysis has been registered in the International Prospective Register of Ongoing Systematic Reviews PROSPERO (registration number: CRD42024562916).

## Introduction

Duchenne muscular dystrophy (DMD) is an inherited X-linked recessive neuromuscular disease, which is caused by a mutations in the DMD gene resulting in deficiency of structural protein myotonic dystrophy proteins (1). The main clinical manifestations are progressive quadriceps lumbar muscle weakness, and loss of the ability to walk by the age of 10-14 years (1). DMD is the most common type of muscular dystrophy, with an incidence rate of about 30 per 100,000 male infants and seems to be the same between regions (1, 2). The condition is marked by a gradual worsening of muscle weakness and atrophy in the trunk and limbs, along with the development of muscle pseudohypertrophy. Additionally, many individuals experience myocardial damage and typically lose the ability to walk by the age of 12 years (3). Due to the absence of a targeted treatment for DMD, the majority of patients typically succumb to respiratory infections and cardiac failure around the age of 20–30 years (4). There have been significant advancements in therapeutic agents for DMD in recent years. These include treatments that address genetic defects (5–7), such as exon skipping, gene replacement therapy, gene editing, and stop codon read-through therapy. Additionally, there are treatments that target the secondary pathological changes of DMD (8, 9), such as glucocorticosteroids, novel anti-inflammatory compounds (vamorolone), inhibitors of muscle growth inhibitors, inhibitors of nuclear factors, cardio-protective compounds, and stem cell therapy. Thus, the natural history of individuals with DMD has undergone significant changes in the last three decades as a result of the adoption of care standards (10).

Over the last decade, clinical studies have found that vamorolone has the same efficacy as glucocorticosteroids in DMD, but lacks the adverse effects associated with glucocorticosteroids (growth retardation, bone disease, muscle atrophy and so on) (11, 12). Glucocorticosteroids and vamorolone are both drugs with pleiotropic effects and multiple mechanisms of action. However, they have distinct differences: vamorolone reduces inflammation without causing the immunosuppressive effects on lymphocyte viability and function that prednisone does (11, 13, 14). A number of animal studies have shown that vamorolone lowered inflammation and enhanced function in mouse models of inflammatory disorders including inflammatory bowel disease, multiple sclerosis, allergic asthma, and myotonic dystrophy (11, 15). First-in-class anti-inflammatory research drug vamorolone has demonstrated safety and efficacy in DMD at 2.0 or 6.0 mg/kg/day (8, 16, 17). Following six months of therapy, the high-dose group (≥2.0 mg/kg/d) in an initial open-label, multiple ascending dose exploratory study in boys with DMD showed notable improvements in motor function (16). Based on the results of multiple studies (8, 16–18), it has been observed that vamorolone at a dosage of 6 mg/kg/day is more effective in maintaining motor outcomes compared to vamorolone at a dosage of 2 mg/kg/day.

Studies have shown that glucocorticosteroids have a notable positive impact on exercise and cardiac function in individuals with DMD. As a result, they are currently recommended for early use in clinical practice. Furthermore, it is suggested that glucocorticosteroids be used to maintain late-stage functional abilities, as well as respiratory and cardiac function after the loss of ambulation (9). Although deflazacort was linked to less weight gain than prednisone, daily use of both drugs is effective in preserving muscle strength for up to 12 weeks (19, 20). Deflazacort offers patients with nonsense mutation DMD clinically significant delays in the loss of physical milestones over 48 weeks when compared to prednisone (21). Although only deflazacort is FDA approved for DMD treatment, both prednisone and deflazacort are used as the standard of care (22–25). But glucocorticosteroids have certain side effects, thus their clinical use is more limited.

There has been controversy regarding the efficacy and safety of different doses of vamorolone in boys with DMD. Currently, there is a lack of data to compare it with deflazacort. In view of this, we conducted a meta-analysis to assess the efficacy and safety of vamorolone at doses of 2.0 or 6.0 mg/kg/day. In addition, we aimed to compare the safety of vamololone with glucocorticosteroids.

### Methods

This study utilized a systematic review and network meta-analysis approach, adhering to the PRISMA guidelines (26). This systematic review and meta-analysis has been registered in the International Prospective Register of Ongoing Systematic Reviews PROSPERO (registration number: CRD42024562916).

### Search strategy

The databases of PubMed, EMBASE and the Cochrane Library were searched from inception to April 2024. We used the following terms: “Vamorolone” OR “Prednisone” OR “Deflazacort” OR “Glucocorticosteroid” OR “Corticosteroid” AND “Duchenne muscular dystrophy.”At least one of the following predetermined outcomes of interest had to be reported by studies: time to stand from supine velocity (TTSTANDV), 6-min walk test distance (6MWD), time to run/walk 10 m velocity (TTRWV), time to climb 4 stairs velocity (TTCLIMBV), and North Star Ambulatory Assessment (NSAA). For the three timed function tests, velocities were computed by dividing 10 by time in seconds (TTRWV) or by taking the reciprocal of the test duration (TTSTANDV, TTCLIMBV). Growth stunting and weight gaining are recognised adverse outcomes associated with glucocorticosteroids, which may be troublesome for boys with DMD during adolescence and is the greatest limitation of current glucocorticosteroids therapy for boys with DMD. Therefore, safety was evaluated by height percentile and BMI z score, which are good indicators of changes in weight and height.

### Article selection and data extraction

The article search was concluded on April, 2024. We selected studies that met the following inclusion criteria: (i) clinical trials; (ii) inclusion of at least one outcome variable; (iii) studies comparing vamorolone, deflazacort, prednisone, orglucocorticosteroids (alone or in combination) for DMD; (iv) the data could be directly obtained or indirectly estimated from figures for statistical analysis.

The exclusion criteria consisted of: (i) duplicate studies; (ii) case reports, reviews, and animal studies; (iii) studies without relevant outcomes; (iv) studies unrelated to the topic; (v) studies published in languages other than English.

Two independent authors (Qin Wang and Yaqing Zeng) reviewed the titles and/or abstracts of the papers that were searched in order to identify appropriate studies. Subsequently, the two authors separately reviewed and modified the complete texts of the obtained reports. The third author (Zhibing Song) resolved any issues that arose among the authors.

We collected the following details: (i) study ID, year of publication, phase of the study, therapy used; (ii) number of patients per arm, age (mean and range) and baseline characteristics. and (iii) outcome data: TTSTANDV or TTSTAND, 6MWD, TTRWV or TTRW, TTCLIMBV or TTCLIMB, BMI z score and Height percentile.

### Quality assessment

The Newcastle Ottawa scale method was employed to evaluate the quality of the primary research (27). Two writers conducted an independent assessment of the quality of the trial’s primary outcome using the Newcastle Ottawa scale (NOS) methodology. This scale was assessed on the basis of individual selection, comparability between groups, and outcome, with a total score of 9 points. Achieving higher scores indicates superior quality in investigations, with a score of 7–9 being seen as indicative of high-quality research. Disputes were settled by an intermediary author.

### Data synthesis

#### Traditional meta-analysis

An analysis was conducted using RevMan software, version 5.4, to assess the effectiveness and safety of vamorolone at different dosages and compare it to Prednisone. The mean difference (MD) of continuous data was reported along with a 95% confidence interval (CI). The heterogeneity test primarily relied on the I^2^ statistic, and the random-effects model was utilized. Results were deemed statistically significant if the *p* < 0.05.

#### Network meta-analysis

To assess the safety of vamorolone in comparison to glucocorticosteroids such as prednisone or deflazacort, a network meta-analysis was conducted on boys with DMD using Stata/MP 18.0. The matrix is represented by the abbreviation HR, and 95% confidence interval (CI) were utilized to assess the significance of differences in effectiveness between each pair of regimens.

## Results

### Study selection

**Figure 1:**
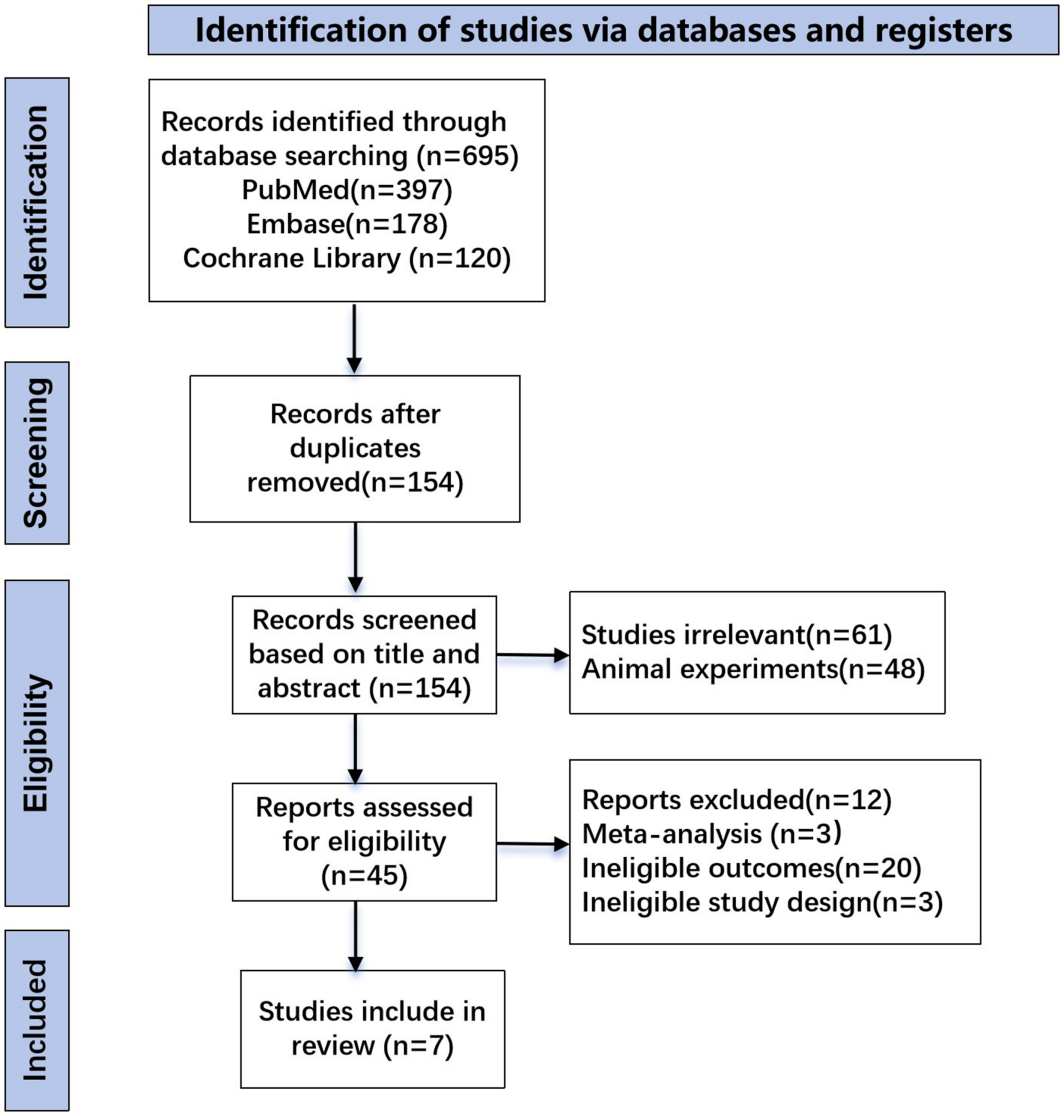
Preferred Reporting Items for Systematic reviews and Meta-Analyses (PRISMA) flow diagram describing the systematic literature search and study selection.

After removing duplicates, we obtained a total of 154 studies. Eventually, seven studies were selected for the analysis (Figure 1). 193 patients of four clinical trials were analyzed in the meta-analysis, including 97 patients receiving vamorolone 2 mg/kg per day and 96 patients receiving vamorolone 2 mg/kg per day. Our network meta-analysis included 363 patients in 4 studies, 53 patients in the deflazacort group, 106 patients in the prednisone group, 129 patients in the vamorolone group, 75 patients in the glucocorticosteroids group. The characteristics of the studies included are reported in Table 1. Table 2 displays the quality of each study that was taken into consideration for analysis. The quality of all studies was good, with a score of 7–8.

**Table 1:** Baseline characteristics.

Study	Year of publication	Study type	Study duration	Drug	Sample size	Age (year)	BMI (kg/m2)	Height percentile	Height (cm)	Weight (kg)	TTSTAND(s)/TTSTANDV (event/s)	TTRW(s)/TTRWV (meters/s)	6MWT (m)	NSAA score	TTCLIMB(s)/TTCLIMBV (event/s)
Shieh et al. (23)	2018	A post hoc analysis	48 weeks	Deflazacort	53	9.2 ± 1.7	18.6 ± 4.7	N/A	127 ± 10.6	30.9 ± 11.9	N/A	N/A	N/A	N/A	N/A
				Prednisone	61	8.8 ± 1.6	19.0 ± 3.5	N/A	125.7 ± 10.4	30.5 ± 9.2	N/A	N/A	N/A	N/A	N/A
Hoffman et al. (16)	2019	An open-label clinical trial	24 weeks	Vamorolone0.25 mg/kg/d	12	5.2 ± 1.0	17.4 ± 1.1	N/A	N/A	N/A	6.1 ± 2.1	6.5 ± 1.4	316 ± 59	19.0 ± 5.1	5.4 ± 1.5
				Vamorolone0.75 mg/kg/d	12	4.8 ± 0.8	16.5 ± 1.5	N/A	N/A	N/A	5.1 ± 2.3	5.9 ± 1.2	331 ± 53	20.5 ± 5.6	4.6 ± 2.8
				Vamorolone2.0 mg/kg/d	12	4.7 ± 0.9	17.2 ± 0.8	N/A	N/A	N/A	5.3 ± 2.0	5.6 ± 1.1	354 ± 65	20.0 ± 4.9	4.6 ± 2.8
				Vamorolone6.0 mg/kg/d	12	4.8 ± 0.8	16.5 ± 1.0	N/A	N/A	N/A	5.9 ± 2.8	6.3 ± 1.2	337 ± 63	19.7 ± 4.9	5.0 ± 2.4
				All vamorolone groups	48	4.9 ± 0.9	16.9 ± 1.2	N/A	N/A	N/A	5.6 ± 2.3	6.1 ± 1.2	334 ± 59	19.8 ± 5.0	4.9 ± 2.4
				Prednisone	14	4.9 ± 0.8	15.2 ± 1.6	N/A	N/A	N/A	5.9 ± 3.2	6.6 ± 2.0	N/A	N/A	6.3 ± 4.1
				Steroid naive	31	5.7 ± 0.7	16.3 ± 1.7	N/A	N/A	N/A	10.03 ± 12.1	6.2 ± 2.4	N/A	N/A	6.1 ± 5.7
Smith et al. (14)	2020	A non-randomized openlabel extension study	18 months	Vamorolone2.0 + 6.0 mg/kg/d	23	5.2 ± 0.9	17.0 ± 0.9	N/A	107 ± 6.8	19.5 ± 2.5	0.206 ± 0.07	1.735 ± 0.331	343.2 ± 64.3	19.9 ± 4.9	0.266 ± 0.134
				Prednisone	12	5.7 ± 0.66	16.5 ± 1.9	N/A	110.3 ± 6.8	20.1 ± 3.5	N/A	N/A	N/A	N/A	N/A
				Corticosteroid-treated	68	5.9 ± 0.64	17.2 ± 1.9	N/A	109.2 ± 5.7	20.6 ± 3.4	N/A	N/A	N/A	N/A	N/A
				Steroid naive	19	5.03 ± 0.55	16.4 ± 0.9	N/A	105.4 ± 5.1	18.3 ± 2.0	0.202 ± 0.055	1.619 ± 0.483	N/A	NA	0.218 ± 0.098
Mah et al. (17)	2022	A nonrandomized controlled trial	30 months	Vamorolone2.0 + 6.0 mg/kg/d	23	5.83 ± 0.88	17.68 ± 1.23	N/A	111.8 ± 6.94	21.98 ± 3.78	0.25 ± 0.10	1.90 ± 0.34	377.9 ± 64.77	23.3 ± 4.72	0.31 ± 0.13
				Corticosteroid-treated	75	6.08 ± 0.81	16.68 ± 1.55	N/A	109.86 ± 6.86	20.35 ± 3.55	0.25 ± 0.10	1.91 ± 0.52	N/A	N/A	0.32 ± 0.14
Guglieri et al. (28)	2022	A randomized, double-blind, placebo clinical trial	24 weeks	Vamorolone2.0 mg/kg/d	30	5.3 ± 0.9	16.2 ± 1.2	30 ± 29	108 ± 9	19 ± 4	0.18 ± 0.05	1.6 ± 0.3	316 ± 58	17.2 ± 4.7	0.21 ± 0.09
				Vamorolone 6.0 mg/kg/d	28	5.4 ± 0.9	16.6 ± 1.4	23 ± 25	107 ± 7	19 ± 3	0.19 ± 0.06	1.6 ± 0.4	313 ± 56	18.9 ± 4.1	0.2 ± 0.05
				Prednisone 0.75 mg	31	5.5 ± 0.9	16.8 ± 1.3	37 ± 29	111 ± 6	21 ± 3	0.22 ± 0.06	1.9 ± 0.4	343 ± 56	21.2 ± 5.5	0.29 ± 0.11
				Steroid naive	28	5.4 ± 0.8	16.3 ± 1.1	33 ± 29	109 ± 9	20 ± 3	0.20 ± 0.06	1.7 ± 0.3	355 ± 78	18.9 ± 5.3	0.25 ± 0.09
Dang et al. (8)	2024	A Randomized controlled Trial	48 weeks	Vamorolone2.0 mg/kg/d	27	5.3 ± 0.9	16.2 ± 1.2	30 ± 29	108 ± 9	19 ± 4	0.18 ± 0.05	1.6 ± 0.3	316 ± 58	17.2 ± 4.7	0.21 ± 0.09
				Vamorolone6.0 mg/kg/d	28	5.4 ± 0.9	16.6 ± 1.4	23 ± 25	107 ± 7	19 ± 3	0.19 ± 0.06	1.6 ± 0.4	313 ± 56	18.9 ± 4.1	0.2 ± 0.05
Leinonen et al. (18)	2023	A randomized and double-blind study	48 weeks	Vamorolone2.0 mg/kg/d	28	5.3 ± 0.9	N/A	43.1 ± 29	N/A	N/A	6.0 ± 2.4	N/A	317 ± 60	17.5 ± 4.6	N/A
				Vamorolone6.0 mg/kg/d	28	5.4 ± 0.9	N/A	43.7 ± 26.7	N/A	N/A	6.0 ± 2.0	N/A	313 ± 56	18.9 ± 4.1	N/A

**Table 2:** Quality assessment for each study considered for analysis.

ID	Selection				Comparability		Outcome			Total point	AHRQ Standards
	(1) Representativeness of the exposed cohort	(2) Selection of the non-exposed cohort	(3) Ascertainment of exposure	(4) Demonstration that outcome of interest was not oresent at start of study	(1) Comparability of cohorts on the basis of design or ananlysis	(2) Comparabiliy of cohorts on the basis of measurement	(1) Assessment of outcome	(2) Was follow-up long enough for outcomes to occur	(3) Adequacy of followup of cohorts		
Shieh et al. (23)	1	1	1	0	0	1	1	1	1	7	Good
Hoffman et al. (16)	1	1	1	0	1	1	1	1	1	8	Good
Smith et al. (14)	1	1	1	0	1	1	1	1	1	8	Good
Mah et al. (17)	1	1	1	0	0	1	1	1	1	7	Good
Guglieri et al. (28)	1	1	1	0	1	1	1	1	1	8	Good
Dang et al. (8)	1	1	1	0	1	1	1	1	1	8	Good
Leinonen et al. (18)	1	1	1	0	1	1	1	1	1	8	Good

### Meta-analysis

#### Vamorolone 6.0 mg/kg/d vs. vamorolone 2.0 mg/kg/d

##### TTSTAND velocity

**Figure 2:**
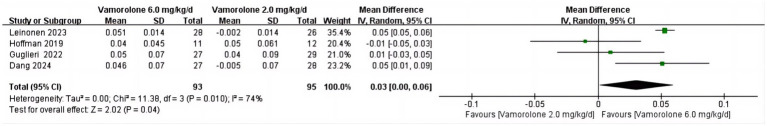
Forest plot of TTSTAND velocity outcome data for vamorolone 6.0 mg/kg/d vs. vamorolone 2.0 mg/kg/d.

Figure 2 presented the forest plot illustrating the velocity change in TTSTAND between vamorolone 6.0 mg/kg/d and vamorolone 2.0 mg/kg/d in each study. Our observation indicates a significant difference in TTSTANDV (MD = 0.03, 95%CI = 0.00–0.06, *p* = 0.04), along with notable heterogeneity among studies (*p* = 0.01, I^2^ = 74%) (Figure 2) (8, 16, 18, 28).

##### TTRW velocity

**Figure 3:**
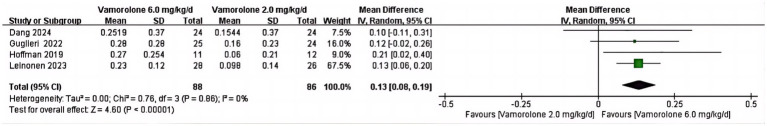
Forest plot of TTRW velocity outcome data for vamorolone 6.0 mg/kg/d vs. vamorolone 2.0 mg/kg/d.

A notable disparity was observed in TTRWV between vamorolone 6.0 mg/kg/d and vamorolone 2.0 mg/kg/d (MD = 0.13, 95%CI = 0.08–0.19, *p* < 0.00001). There was no significant heterogeneity among the studies (*p* = 0.86, I^2^ = 0%) (Figure 3) (8, 16, 18, 28).

##### 6MWT

**Figure 4:**
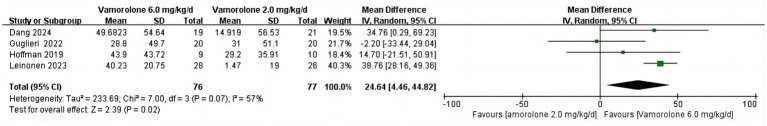
Forest plot of 6MWT velocity outcome data for vamorolone 6.0 mg/kg/d vs. vamorolone 2.0 mg/kg/d.

The pooled analysis showed a significant difference between vamorolone 6.0 mg/kg/d and vamorolone 2.0 mg/kg/d in 6MWT (MD = 24.54, 95%CI = 4.46–44.82, *p* = 0.02), and a significant heterogeneity among studies (*p* = 0.07, I^2^ = 57%) (Figure 4) (8, 16, 18, 28).

##### TTCLIMB velocity

**Figure 5:**
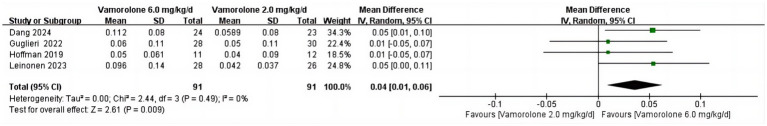
Forest plot of TTCLIMB velocity outcome data for vamorolone 6.0 mg/kg/d vs. vamorolone 2.0 mg/kg/d.

The combined analysis revealed no significant heterogeneity across studies (*p* = 0.49, I^2^ = 0%) and a significant difference in TTCLIMBV between vamorolone 6.0 mg/kg/d and vamorolone 2.0 mg/kg/d (MD = 0.04, 95%CI = 0.01–0.06, *p* = 0.009) (Figure 5) (8, 16, 18, 28).

##### BMI z score

**Figure 6:**
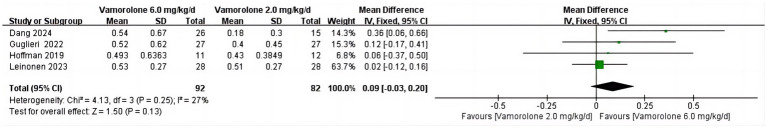
Forest plot of BMI z score outcome data for vamorolone 6.0 mg/kg/d vs. vamorolone 2.0 mg/kg/d.

We found no statistically significant difference in BMI z score between vamorolone 2.0 mg/kg/d and vamorolone 6.0 mg/kg/d (MD = 0.09, 95%CI = −0.03–0.20, *p* = 0.13) or significant heterogeneity amongst studies (*p* = 0.25, I^2^ = 27%) (Figure 6) (8, 16, 18, 28).

#### Vamorolone vs. prednisone

##### BMI z score

**Figure 7:**
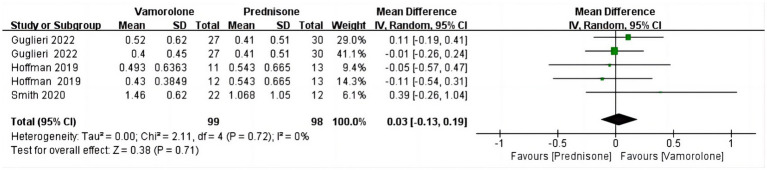
Forest plot of BMI z score outcome data for vamorolone vs. prednisone.

The combined analysis revealed no statistically significant difference in the BMI z score between vamorolone and prednisone (MD = 0.03, 95%CI = −0.13–0.19, *p* = 0.71) or in the heterogeneity among the studies (*p* = 0.72, I^2^ = 0%) (Figure 7) (14, 16, 28).

##### Mean height percentile for age

**Figure 8:**
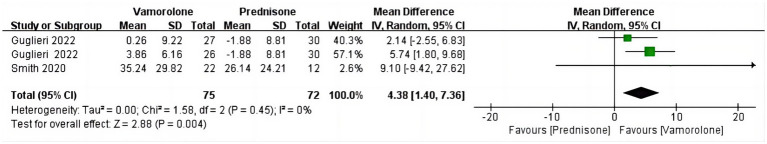
Forest plot of mean height percentile for age outcome data for vamorolone vs. prednisone.

The pooled analysis revealed a statistically significant disparity in the Mean height percentile for age between vamorolone and prednisone (MD = 4.38, 95%CI = −1.40–7.36, *p* = 0.004). There was no significant variation among the studies (*p* = 0.45, I^2^ = 0%) (Figure 8) (14, 28).

#### Network meta-analysis

##### BMI z score

**Figure 9:**
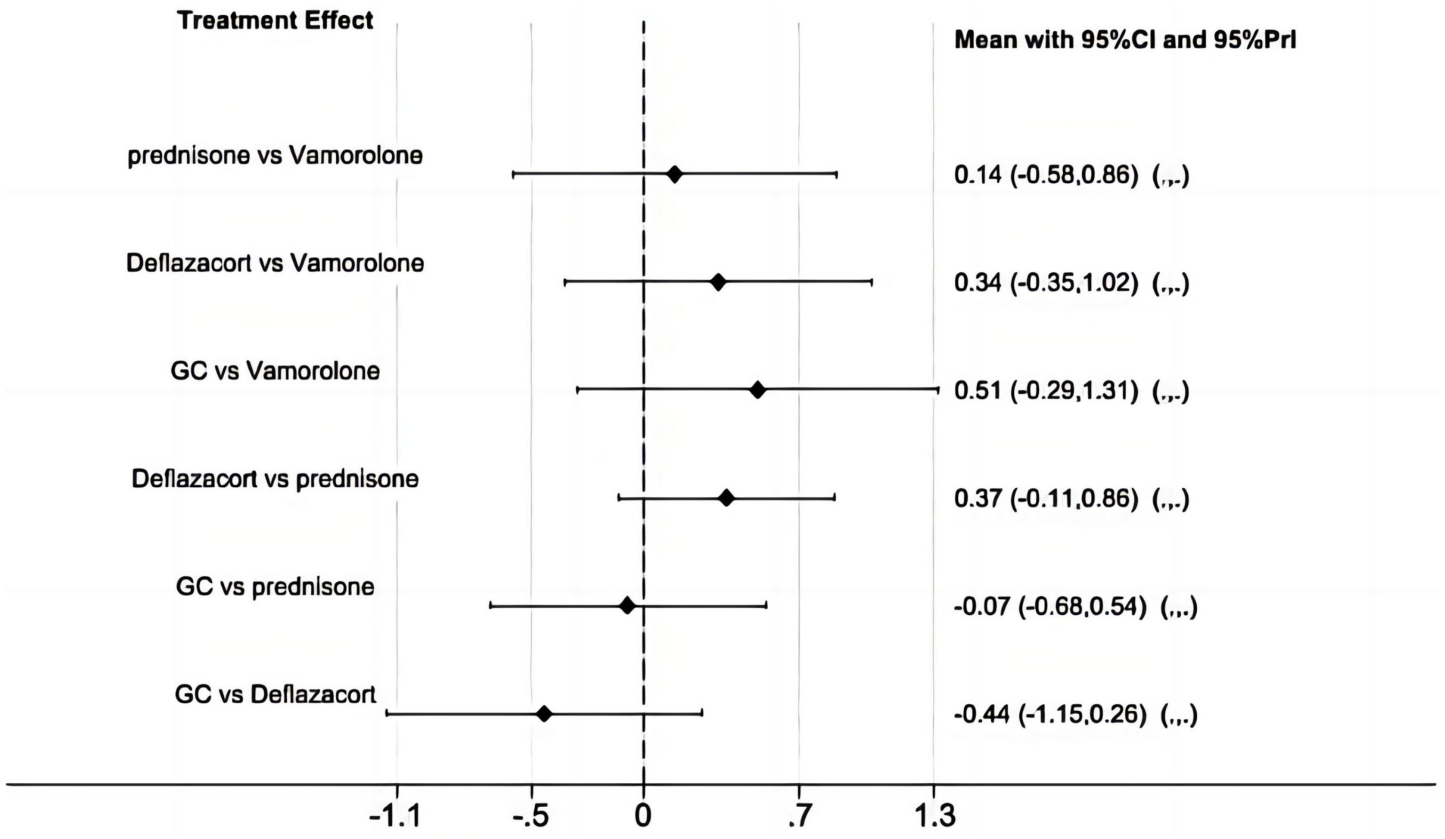
Network meta-analysis for vamorolone vs. glucocorticosteroid, prednisone and deflazacort of BMI z score.

The network meta-analysis did not find any significant differences in BMI z score among vamorolone, glucocorticosteroid, prednisone, and deflazacort, based on indirect comparisons (Figure 9) (16, 17, 23, 28).

## Discussion

To our knowledge, this is the first meta-analysis to compared vamorolone 6 mg/kg per day with vamorolone 2 mg/kg per day in boys with DMD. From the results of our study, we conclude DMD patients may have greater response to vamorolone 6 mg/kg per day without causing weight gain.

A new drug called vamorolone seems to maximize conventional steroidal anti-inflammatory effects by retaining transrepression (NF-κB inhibition anti-inflammatory activity) and losing transactivation (gene transcription via GRE-mediated binding of ligand/receptor dimers) (11, 13). Comparing vamorolone to prednisolone, studies in animal models of chronic inflammatory states, including DMD mouse models, have demonstrated retention of anti-inflammatory efficacy and reduction of side effects (11, 12, 29). Similar to glucocorticosteroids in pharmacokinetics and metabolism, vamorolone is given orally once a day (30, 31). In contrast to glucocorticosteroids, vamorolone does not interact with 11 β-hydroxysteroid dehydrogenase regulatory enzymes and effectively blocks the mineralocorticoid receptor (15). Vamorolone has demonstrated efficacy in preserving muscle function and extending walking time in patients with DMD, as evidenced by multiple clinical trials (13, 14). Consequently, it represents a promising and groundbreaking therapeutic option for the treatment of DMD (32). Vamorolone received approval in October 2023 for treating DMD in patients aged 2 and older in the USA. It also received a positive opinion in the EU in October 2023 for treating DMD in patients aged 4 and older (33). However, the specific dosage remains uncertain, as it is only indicated that a dose of 2-6 mg/kg might be suitable.

Boys with DMD who received vamorolone 6 mg/kg/day compared to vamorolone 2 mg/kg/day improved in several functional end points, including TTSTANDV, TTRWV, 6MWT and TTCLIMBV, according to this meta-analysis. But BMI z score is not statistically significant. Our results support the conclusions of Dang et al. (8), who found that for most motor outcomes, vamorolone at a dose of 6 mg/kg/d demonstrated better maintenance of effect than vamorolone at a dose of 2 mg/kg/d. Like us, Leinonen et al. (18) found that in TTSTAND velocity, the effect observed at week 24 for vamorolone 6 mg/kg/day persisted until week 48, but not for vamorolone 2 mg/kg/day at week 48. That study also revealed that, compared to TTRW or NSAA, DMD patients taking 6 mg/kg of vamorolone daily improved more in 6MWT and TTCLIMB. Hoffman et al. (16) discovered that vamorolone 6.0 mg/kg/d improved 6MWT and TTRW more than 2.0 mg/kg/d, but this dose also raised more safety concerns, with a mean rise in BMI z score comparable to that with prednisone. Although Guglieri et al. (28) found that the variations in TTSTANDV and, 6MWT were clinically significant, this study did not compare the functional end points of vamorolone 6 mg/kg daily with vamorolone 2 mg/kg daily.

During the initial investigation into the exposure-response relationships of vamorolone, it was discovered that significant enhancements in clinical efficacy were observed at a daily dose of 2 mg/kg to 6 mg/kg after 24 weeks. A study found that higher-dose groups (≧2.0 mg/kg/d) experienced significant motor function improvement after 6 months of treatment (14). Additionally, the long-term results of this study revealed that vamorolone was associated with the maintenance of muscle strength and function for up to 30 months. This effect was similar to standard-of-care glucocorticoid therapy. Furthermore, vamorolone showed improved height velocity compared to the growth deceleration observed with glucocorticoid therapy. These findings suggest that vamorolone may be a promising option for the treatment of DMD (17). Significant increases in TTSTAND, TTRW, and TTCLIMB were observed in a meta-analysis of vamolodone versus placebo and steroids, which suggests that vamolodone has benefits in the treatment of DMD patients (34). Thus, our combined analysis and the studies mentioned above suggest that boys with DMD who took vamorolone 6 mg/kg daily may have better motor function than those who took vamorolone 2 mg/kg daily and that there is no appreciable difference in the impact on body weight. Unfortunately, the studies we included were unable to access the mean height percentile for age in boys with DMD, who recieved vamorolone 6 mg/kg/day or vamorolone 2 mg/kg/day.

Administering glucocorticosteroids to children with DMD can enhance muscle mobility and postpone damage to the heart muscle, resulting in a longer period of walking and ultimately improving the survival rate of patients (22, 24). Glucocorticosteroids, which are commonly used to treat children with DMD, have been found to cause several negative effects. These include stunted growth, hormonal imbalances, weakened bones, metabolic disorders, increased body weight, and delays in puberty development (9, 19, 25). More previous studies have identified two measures of safety concerns related to glucocorticoids: growth deceleration (growth retardation measured as change in mean height-for-age percentile) and body mass index (BMI). In children, chronic glucocorticosteroids treatment often results in growth deceleration (8, 9, 14, 17). Vamorolone and prednisone differed significantly in the mean height percentile for age in the pooled analysis. Like us, Hoffman et al. (16) found that boys with DMD who took vamorolone 6 mg/kg daily had a mean rise in BMI z score comparable to that of prednisone. However, our findings indicated that vamorolone has little effect on bone formation. Mah et al. (17) reported similar findings to ours, showing that vamorolone increased height velocity in contrast to growth deceleration associated with glucocorticoid treatment. Dang et al. (8) further found that switching from prednisone to vamolone therapy reversed the bone morbidity of prednisone (growth retardation and decreased serum bone biomarkers). The Guglieri study also revealed the analogous results that height percentile decreased in participants receiving prednisone (but not vamorolone) (28). It can be concluded from these studies and our meta-analysis that vamorolone has a far smaller impact on height development than glucocorticoid.

Glucocorticosteroids are utilized as the standard treatment for DMD. However, it is important to note that only deflazacort has received FDA approval specifically for DMD (35). We conducted a safety comparison indirectly between vamorolone, glucocorticosteroid, prednisone, and deflazacort. However, our findings indicate that there are no significant differences in BMI z score among these medications. More trials are needed to explore the safety comparisons among vamorolone, glucocorticosteroid, prednisone, and deflazacort.

### Future implications

The results of our meta-analysis suggest that DMD boys taking 6 mg/kg per day instead of 2 mg/kg per day vamorolone may be more advantaged in timed functional tests like TTSTANDV, TTRWV, TTCLIMBV and 6MWT, without affecting BMI. Our study suggests that DMD boys taking 6 mg/kg of vamorolone per day may have greater improvement in motor function. However, safety studies on 2 mg/kg per day vamorolone and 6 mg/kg per day vamorolone are still relatively scarce and need to be further explored. Due to the fact that there are no studies with larger doses, while whether there is a better effect at doses higher than 6 mg/kg per day vamorolone still needs to be discovered in more studies.

### Strengths and limitations

This meta-analysis compared the efficacy and safety of different doses of vamorolone, and safety of vamorolone vs. glucocorticosteroid, prednisone or deflazacort in boys with DMD. But this study has several limitations. First, BMI z score comparisons between vamorolone, glucocorticosteroid, predinisone or deflazacort in this meta-analysis were indirect. And we failed to analyse comparative data on motor function between vamorolone and glucocorticoids, due to differences in study evaluations. Second, since there was no information in the current studies about comparison of height percentile between the 2 mg/kg and 6 mg/kg dose, this meta-analysis did not compare vamorolone 6 mg/kg per day compared with vamorolone 2 mg/kg per day in mean height percentile for age. Third, some of our studies were clinical trials but not randomized. In addition, the study periods and age ranges of the study population are not the same, because of limitations on the number of studies.

## Conclusion

Our study indicates that TTSTANDV, TTRWV, 6MWT, and TTCLIMBV of boys with DMD who received vamorolone 6 mg/kg daily improved better compared to vamorolone 2 mg/kg daily. But the BMI z score is not statistically significant. Boys with DMD who take vamorolone 6 mg/kg daily may have better function and be safer. But more large sample size randomized clinical trials are required to investigate the long-term safety and effectiveness of various vamorolone dosages.

## Data Availability

The original contributions presented in the study are included in the article/supplementary material, further inquiries can be directed to the corresponding author.

## Glossary

6MWD: 6-minute walk distance
BMI: Body mass index
DMD: Duchenne muscular dystrophy
TTCLIMB: Time to climb 4 steps
NSAA: North Star Ambulatory Assessment
TTRW: Time to run/walk 10 m
TTSTAND: Time to stand from supine
TTCLIMBV: Time to climb 4 steps velocity
TTRWV: Time to run/walk 10 m velocity
TTSTANDV: Time to stand from supine velocity
